# Guillain-Barré Syndrome Following Respiratory Syncytial Virus (RSV) Infection in the Third Trimester: A Case Report of a Rare Entity

**DOI:** 10.7759/cureus.105685

**Published:** 2026-03-23

**Authors:** Falak Naz Baloch, Humna Mian Fiaz Rasul, Nicola Roberts, Liliana Grosu

**Affiliations:** 1 Department of Obstetrics and Gynaecology, Bedfordshire Hospitals NHS Foundation Trust, Bedford, GBR

**Keywords:** guillain-barre syndrome, immune-mediated peripheral neuropathies, intravenous immunoglobulin (ivig), polyradiculoneuropathy, pregnancy, respiratory syncytial virus (rsv) vaccine, third trimester of pregnancy

## Abstract

Guillain-Barré syndrome (GBS) is an acute immune-mediated polyradiculoneuropathy affecting the peripheral nervous system, with a range of clinical presentations. GBS in pregnancy is rare and creates diagnostic and management challenges, with limited evidence to guide decisions on the timing and mode of delivery. We report a case of a 37-year-old gravida 4, para 3, who presented at 34+3 weeks of gestation with progressive lower limb weakness, sensory loss, and urinary incontinence. She had received a respiratory syncytial virus (RSV) vaccine shortly before the onset of symptoms. Neurological evaluation, including lumbar puncture and nerve conduction studies, supported a diagnosis of GBS with cranial nerve involvement. The patient was managed with intravenous immunoglobulin (IVIG), physiotherapy, and coordinated multidisciplinary obstetric and anesthetic care. She underwent induction of labor at 37+2 weeks and achieved a spontaneous vaginal delivery of a healthy infant. After delivery, maternal neurological function improved with ongoing physiotherapy. This report highlights the importance of early recognition of GBS in pregnancy, the role of a multidisciplinary team (MDT) in management, and the feasibility of vaginal delivery in appropriately selected patients.

## Introduction

Guillain-Barré syndrome (GBS) comprises a spectrum of immune-mediated peripheral neuropathies that most commonly affect the myelin sheath of peripheral nerves, leading to varied clinical presentations [1,2]. It is one of the leading causes of acute flaccid paralysis worldwide, with an incidence of approximately one to two cases per 100,000 people annually [3]. While its overall incidence in pregnancy is similar to that in the general population, women are especially susceptible during the third trimester and early postpartum period because of immunological changes [4]. Infections with pathogens such as Campylobacter jejuni, Zika virus, and respiratory viruses, including influenza and severe acute respiratory syndrome coronavirus 2 (SARS-CoV-2), can trigger an immune response through the production of autoantibodies [5]. Post-marketing surveillance has identified a rare but notable risk of GBS after respiratory syncytial virus (RSV) vaccination, particularly in older adults, highlighting the importance of ongoing safety monitoring and risk-benefit assessment [6].

GBS may develop at any stage of pregnancy or during the postpartum period, with the highest incidence observed in the third trimester and within the first two weeks after delivery [7]. GBS during pregnancy is uncommon, with an annual incidence of approximately 2.8 cases per 100,000 individuals. Pregnancy is already associated with significant physiological changes and potential complications, and the occurrence of GBS further complicates maternal and fetal management during the peripartum period [8]. Although GBS in pregnancy is rare, it is associated with increased maternal and perinatal morbidity and carries a risk of mortality [9].

The clinical presentation of GBS varies depending on the subtype. Progressive weakness is the most frequent symptom, typically beginning in the lower extremities and ascending toward the upper body. Early manifestations often include difficulty walking and maintaining balance, and by the third week after symptom onset, approximately 90% of patients reach their maximum level of weakness [10]. Consequently, GBS may present with a wide range of nonspecific symptoms, often leading to delays in diagnosis or misdiagnosis. We report a case of GBS in pregnancy that was initially misdiagnosed due to an atypical presentation [11].

## Case presentation

A 37-year-old multigravida (G4P3) of Pakistani ethnicity presented to the emergency department at 34+3 weeks’ gestation with progressive neurological symptoms. Her complaints included worsening lower limb numbness, bilateral weakness, unsteady gait, urinary incontinence, loss of sensation from the hips downward, and paraesthesia in the fingers. There was no history of trauma or similar previous episodes.

Her medical history was significant for hypothyroidism, managed with levothyroxine 50 micrograms daily. She had no known drug allergies, was a non-smoker (carbon monoxide level 1), and had a booking BMI of 23 kg/m². Family history was notable for type 2 diabetes mellitus in her mother. Her venous thromboembolism (VTE) risk assessment at booking indicated she was not at high risk, and pharmacological thromboprophylaxis with tinzaparin was not required. Patient demographics, clinical presentation, investigations, management, and outcomes are summarised in Table 1.

**Table 1:** Patient summary: demographics, clinical features, investigations, management, and outcomes This table summarises the key clinical details of the patient, including demographic information, gestational age at presentation, presenting symptoms, relevant medical and obstetric history, major investigations, treatment interventions, delivery details, and maternal and neonatal outcomes. The table provides a concise overview of the case, highlighting the complexity of managing Guillain–Barré syndrome in late pregnancy and the multidisciplinary approach required for optimal outcomes SVD: spontaneous vaginal delivery; SGA/FGR: small for gestational age/fetal growth restriction; MRI: magnetic resonance imaging; LP: lumbar puncture; NCS: nerve conduction studies; IVIG: intravenous immunoglobulin; MDT: multidisciplinary team

Parameter	Details
Age/parity	37 years, G4P3 (all SVD)
Gestation at presentation	34+3 weeks
Symptoms	Lower limb weakness, numbness, urinary incontinence, and facial nerve involvement
Medical history	Hypothyroidism, previous SGA/FGR pregnancies
Investigations	MRI spine: normal; LP: raised protein; NCS: facial nerve axonal impairment
Management	IVIG, physiotherapy, MDT care, thromboprophylaxis
Delivery	Induction at 37+2 weeks, vaginal delivery, birthweight 1840 g, Apgar 9/10 [12]
Outcome	Maternal neurological improvement, healthy newborn

Her antenatal course had been uncomplicated until presentation. Due to a history of small-for-gestational-age and fetal growth restriction in previous pregnancies, she underwent enhanced fetal surveillance. Booking investigations were normal, including blood group A positive with no atypical antibodies, normal blood pressure, and planned oral glucose tolerance testing at 26-28 weeks. The anomaly scan at 20+3 weeks, and uterine artery Doppler studies were normal. A growth scan at 32+2 weeks showed an estimated fetal weight on the 87.2nd centile with normal liquor volume and umbilical artery Dopplers. She received the RSV vaccination and subsequently developed a confirmed RSV infection approximately 12 days later. Four days after the diagnosis, she was admitted with acute neurological deterioration, including lower limb weakness, unsteady gait, and urinary incontinence.

Neurological examination revealed a Glasgow Coma Scale (GCS) score of 15/15, which is a standard, freely available tool for assessing consciousness, along with stable vital signs [13]. Motor power below the knees was reduced to 2-3/5 bilaterally, with absent sensation below the knees. During admission, she developed new-onset right-sided facial weakness consistent with a lower motor neuron facial nerve palsy, for which corticosteroids were commenced. MRI of the lumbar spine was unremarkable. Cerebrospinal fluid analysis following lumbar puncture demonstrated raised protein levels with otherwise normal parameters, consistent with albuminocytologic dissociation (ACD). Nerve conduction studies revealed axonal impairment of the seventh cranial (facial) nerve with reduced left nasalis compound muscle action potential, along with bilateral carpal tunnel syndrome (more severe on the right). There was no electrophysiological evidence of acute inflammatory demyelinating polyneuropathy (AIDP). Ophthalmological assessment showed no Kayser-Fleischer rings, effectively excluding Wilson’s disease.

Given the clinical presentation and investigation findings, a diagnosis of GBS with cranial nerve involvement was made. The diagnosis of GBS was established using the Brighton diagnostic criteria for GBS, a freely available tool widely used to standardise case definitions [14]. Throughout her hospital stay, she reported reduced perception of fetal movements, necessitating daily cardiotocography from 33+6 weeks’ gestation, all of which were reassuring. At 36+2 weeks, ultrasound demonstrated a reduced growth velocity, with estimated fetal weight declining to the 35th centile but with normal liquor volume and Doppler indices.

A multidisciplinary team (MDT) discussion involving obstetrics, neurology, anaesthetics, maternal medicine (Addenbrooke’s Hospital), physiotherapy, and neonatology concluded that early delivery was not indicated solely due to GBS in the absence of respiratory or bulbar compromise. Conservative management was recommended, with escalation only if rapid neurological deterioration occurred. The patient received a five-day course of intravenous immunoglobulin (IVIG) (Gamunex 10%, 20 g daily), commenced shortly after admission. A peripherally inserted central catheter was placed for prolonged intravenous access. She underwent intensive physiotherapy and dietetic support following unintentional weight loss. Thromboprophylaxis with tinzaparin was administered and later withheld before delivery.

At 37+2 weeks’ gestation, the patient was transferred to the delivery suite for induction of labour. Continuous fetal monitoring was normal. She progressed rapidly and achieved spontaneous vaginal delivery of a live female infant weighing 1840 g (42.3rd centile). Neonatal status was assessed using the Apgar score, a standard and freely available tool, with scores of 9 at one minute and 10 at five minutes [12]. The third stage of labour was actively managed, with an estimated blood loss of 150 mL. She sustained a first-degree perineal tear, which was sutured without complication.

Postnatally, her neurological function improved steadily. By day seven, she was mobilising with the aid of a walking stick. She was treated for a urinary tract infection and continued on thromboprophylaxis. The PICC line was removed, and she was discharged home with community midwife follow-up and physiotherapy support. Comprehensive laboratory, cerebrospinal fluid, imaging, and microbiological investigations are summarised in Table 2.

**Table 2:** Laboratory, CSF, imaging, and microbiological investigations of the patient This table summarizes the patient’s laboratory, CSF, imaging, and microbiological investigations, including reference ranges and brief interpretations. Abnormal findings relevant to the diagnosis of Guillain-Barré syndrome, liver enzyme trends, and recent RSV infection are highlighted, while other investigations were within normal limits CSF: cerebrospinal fluid; ALT: alanine aminotransferase; MRI: magnetic resonance imaging; RBC: red blood cells; AFP: alpha-fetoprotein; EBV: Epstein Barr virus; IgG: immunoglobulin G; CMV: cytomegalovirus; Hep A: hepatitis A; HBsAg: hepatitis B surface antigen; HBcAg: hepatitis B core antigen; PCR: polymerase chain reaction; RSV: respiratory syncytial virus; MRSA: methicillin-resistant Staphylococcus aureus

Investigation	Result	Reference range	Interpretation
ALT (U/L)	62 → 77 → 90 → 134 → 184 → 277 → 457 → 524 → 714 → 666 → 730 → 792 → 813 → 756 → 744 → 655 → 611 → 448 → 384 → 201	7–56	Rapid rise with peak 813 U/L, gradual normalization
Bile acids (µmol/L)	4	0–10	Normal
Liver ultrasound	Normal	—	No structural abnormality
MRI whole spine	Normal	—	No disc disease or neural compromise, excludes transverse myelitis
CSF protein (g/L)	0.5	0.15–0.45	Elevated (albuminocytologic dissociation)
CSF glucose (mmol/L)	3.9	2.2–4.4	Normal
CSF WBC (cells/mm³)	<5	0–5	Normal
CSF RBC (cells/mm³)	<5	0	Normal
CSF cultures	No growth at 48 hrs	—	Normal
AFP (ng/mL)	253	<400	Normal
Viral serology	EBV IgG/IgM, CMV IgG/IgM, Hep A and E IgM, HBsAg, HBcAg	Negative	No evidence of active infection
CMV PCR	Not detected	—	Negative
Influenza RNA A/B	Not detected	—	Negative
RSV RNA	Detected	—	Recent RSV infection
Copper (µmol/L)	35.1	12–25	Mildly elevated
Ceruloplasmin (g/L)	0.48	0.2–0.4	Mildly elevated
MRSA culture	Not detected	—	Negative
Nerve conduction studies	Axonal impairment of CN VII, bilateral carpal tunnel (R>L)	—	Supports GBS diagnosis

The key events, investigations, and interventions during the patient’s clinical course are summarised in Table 3. Key findings included elevated CSF protein consistent with albuminocytologic dissociation and nerve conduction study abnormalities supporting the diagnosis of GBS, while liver enzymes showed a transient rise in ALT.

**Table 3:** Timeline of clinical events, investigations, management, and delivery outcomes This table provides a chronological overview of the patient’s clinical course, beginning with the RSV vaccination and infection, followed by symptom onset, hospital admission, neurological investigations (lumbar puncture and nerve conduction studies), therapeutic interventions (intravenous immunoglobulin and physiotherapy), delivery, and postnatal outcomes. It highlights the temporal relationship between key events and management decisions, illustrating the complexity of caring for Guillain–Barré syndrome in late pregnancy and the multidisciplinary approach undertaken RSV: respiratory syncytial virus; IVIG: intravenous immunoglobulin

Gestation	Event
33+0	RSV vaccination
33+2	RSV infection
34+3	Admission with weakness
35+3	Lumbar puncture
35+5	Nerve conduction studies
36+0	Start IVIG
37+2	Transfer to the delivery suite
37+2	Spontaneous vaginal delivery

## Discussion

The incidence of GBS in pregnancy is similar to that in non-pregnant women and ranges from 1.2 to 1.9 cases per 100,000 annually [1]. GBS most commonly occurs in the third trimester of pregnancy and within the first two weeks postpartum; however, it can occur in any trimester and at any time during the postpartum period [1]. Due to the rapid decrease in delayed-type hypersensitivity in the postpartum period, GBS is known to worsen postpartum [15]. In a literature review of 30 cases of GBS in pregnancy, onset occurred in the first trimester in four women, in the second trimester in 14 women, and in the third trimester in 12 women [16]. This highlights that GBS can occur throughout pregnancy, necessitating vigilance among clinicians across all trimesters. GBS is frequently precipitated by infections, but it may also follow physical trauma, vaccination, or other immune-mediated events. In up to 30% of cases, no clear trigger can be identified [17]. Approximately two-thirds of patients report a preceding infectious illness, most commonly an acute viral respiratory infection (40%) or a gastrointestinal infection (20%), occurring four to six weeks before symptom onset [18].

Neurological assessment typically reveals weakness, paralysis, sensory disturbances, and altered reflexes, whereas autonomic involvement may present with cardiovascular, respiratory, or gastrointestinal symptoms [1]. Although fetal development is generally unaffected, maternal complications can be significant, and progression of neurological symptoms may necessitate urgent delivery. Current literature suggests that GBS does not adversely affect fetal development or increase the risk of spontaneous abortion; however, the progression of maternal neurological symptoms may necessitate urgent delivery. A reported case of GBS at 33 weeks’ gestation, initially misdiagnosed as a viral illness, demonstrated worsening bulbar involvement despite treatment, ultimately requiring an emergency cesarean delivery [1,10]. Furthermore, GBS is associated with a significant risk of maternal neuromuscular respiratory failure, with up to 34.5% of affected pregnant women requiring ventilatory support [18]. Patients exhibiting signs of respiratory failure, such as dyspnea, tachypnea, abnormal arterial blood gases, or use of accessory muscles, should be closely monitored and considered for ICU admission [10].

Although the exact pathogenesis of GBS remains unclear, it is believed to result from molecular mimicry between gangliosides in Schwann cells and epitopes on the cell walls of certain microorganisms [10]. Because GBS often presents with non-specific symptoms such as pain, weakness, numbness, and paresthesia, diagnosis during pregnancy is frequently delayed, as these features may overlap with common pregnancy-related changes. Differential diagnoses include sciatica, carpal tunnel syndrome, pregnancy-related musculoskeletal changes, transverse myelitis, myasthenia gravis, botulism, and Lyme disease [10].

Standardised diagnostic frameworks such as the Brighton criteria are valuable to support timely diagnosis and management. The Brighton criteria provide a standardised framework, incorporating exclusion of alternative causes of weakness, hypo- or areflexia, a monophasic course, bilateral flaccid limb weakness, elevated CSF protein with normal cell counts, and supportive findings on nerve conduction studies. Early recognition using these criteria, along with vigilant monitoring for respiratory or autonomic complications, is critical to reduce maternal morbidity and mortality [14]. A hallmark feature of GBS is elevated CSF protein with a normal white blood cell count, known as ACD, which reflects disruption of the blood-nerve barrier, increased intrathecal antibody production, or both [19]. According to Wang et al. (2023), when imaging and laboratory findings are inconclusive, careful monitoring of clinical progression and CSF analysis is essential for prompt diagnosis [20]. These diagnostic strategies are particularly important given the variable timing of onset of GBS in pregnancy, emphasising the need for a high index of suspicion. Literature reviews of GBS in pregnancy show variable timing of onset, with cases reported across all trimesters, highlighting the importance of maintaining a high index of suspicion.

In non-ambulatory patients, GBS is treated with immunotherapy, either IVIG or plasma exchange (PLEX). Both therapies are considered safe and effective for improving muscle strength and reducing the need for mechanical ventilation in severe cases [10]. Functional recovery following GBS is typically gradual, with approximately 80% of patients regaining independent ambulation and over half achieving full recovery within one year [10].

## Conclusions

GBS in pregnancy is rare but may result in significant maternal morbidity and fetal risks, particularly when presenting with atypical features such as cranial nerve involvement. Early recognition, prompt investigations, and multidisciplinary management are essential to optimise outcomes. This report illustrates that with coordinated care, conservative management, and careful delivery planning, favourable maternal neurological recovery and healthy neonatal outcomes are achievable, even in late pregnancy complicated by GBS. Reporting such cases adds to the limited evidence base and guides clinicians managing similar complex scenarios.
